# Molecular and cellular mechanisms underlying postoperative paralytic ileus by various immune cell types

**DOI:** 10.3389/fphar.2022.929901

**Published:** 2022-08-03

**Authors:** Chao Sui, Liang Tao, Chunhua Bai, Lihua Shao, Ji Miao, Kai Chen, Meng Wang, Qiongyuan Hu, Feng Wang

**Affiliations:** ^1^ Department of Gastrointestinal Surgery, Nanjing Drum Tower Hospital, The Affiliated Hospital of Nanjing University Medical School, Nanjing, China; ^2^ Medical School of Nanjing University, Nanjing, China

**Keywords:** postoperative ileus, inflammatory response, immune cell, macrophage, neutrophil, mast cell

## Abstract

Postoperative ileus (POI) is a well-known complication following gut manipulation or surgical trauma, leading to an impaired gut motility and prolonged postoperative recovery time. Few current therapeutic strategies can prevent POI, and this disorder remains to be a major clinical challenge for patients undergoing surgery. Comprehensive understanding of cellular and molecular mechanisms related to the pathogenesis of POI stimulates the discovery of more promising targets for treatment. POI is closely associated with a series of inflammatory events within the bowel wall, and as key components of inflammatory mechanisms, different types of immune cells, including macrophages, dendritic cells, and T lymphocytes, play significant roles during the development of POI. A variety of immune cells are recruited into the manipulation sites after surgery, contributing to early inflammatory events or impaired gut motility. Our review intends to summarize the specific relationship between different immune cells and POI, mainly focusing on the relevant mechanisms underlying this disorder.

## Introduction

**FIGURE 1:**
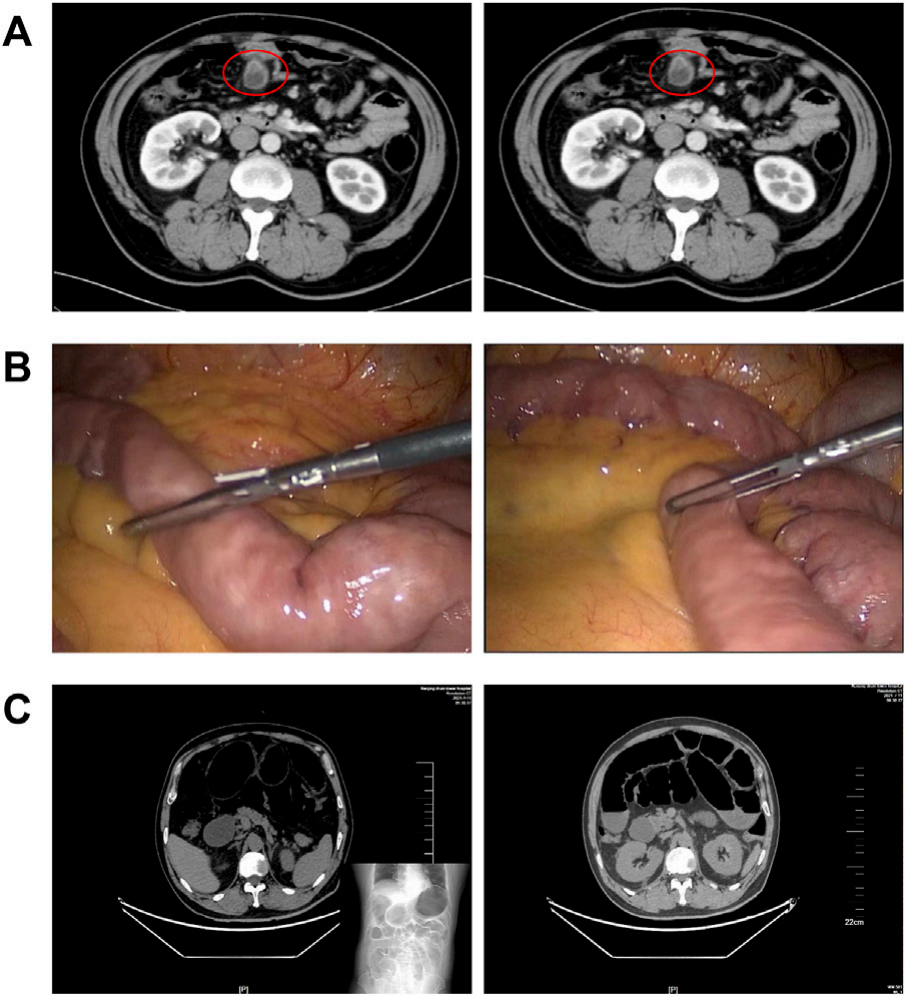
Gut manipulation in the abdominal surgery leads to postoperative ileus. **(A)** CT imaging features of a patient with small bowel diverticulum (red circles); **(B)** photos of intestinal manipulation during endoscopic surgery of this patient; and **(C)** postoperative imaging suggested that this patient had severe ileus.

Postoperative ileus (POI), characterized by a transient cessation of gastrointestinal (GI) function, is a common complication following general surgery or gut manipulation (Behm and Stollman, 2003; Hedrick et al., 2018; Thomas, 2019). This clinical dilemma has been a considerable burden on both inpatients and medical resources because of prolonged hospitalization time and increased expenses (Ramirez et al., 2013; Bragg et al., 2015; Wolthuis et al., 2016). Figure 1 presents us the imaging features of a patient with severe POI. Intraoperative intestinal manipulation directly led to the generation of POI, which subsequently resulted in poor prognosis.

Currently, POI is considered to be mainly related to sympathetic neural reflexes, activation of gut opioid receptors and inflammatory reaction, which eventually lead to symptoms such as vomiting, abdominal distension, and delay of defecation in patients (Vather et al., 2013; Vather et al., 2014; Wattchow et al., 2021). Recent evidence suggested that different types of immune cells play a vital role in the genesis of POI (Wattchow et al., 2021), and studies based on it are trying to find a novel potential target for treatment (Mazzotta et al., 2020). However, the relationship between immune cells and POI lacks further investigation and systemic summary. This review intends to provide a general understanding of the mechanisms under POI. It draws together information on effects of various immune cells on paralytic ileus, covering monocytes, macrophages, neutrophils, dendritic cells, mast cells, and T lymphocytes. Other important contributors are also briefly summarized in our review, with emphasis on molecular and cellular mechanisms.

## Mechanisms under postoperative ileus

Several studies have focused on the pathophysiological process of POI in the past few years. Neurogenic as well as inflammatory mechanisms are considered to be mainly involved in the pathogenesis processes of this disorder (Wehner et al., 2012). It is now widely accepted that a neurogenic component plays a significant role in the early phase of postoperative impairment of gut motility (Lubbers et al., 2010). Activation of sympathetic pathways in response to surgical trauma or gut manipulation is verified to mediate a widespread inhibition of GI function, mostly through suppression of enteric neural reflex pathways (Stakenborg et al., 2017a).

The second phase of ileus is related to an immunological and inflammatory response that consequently leads to a prolonged duration of POI (Venara et al., 2016). Previous evidence in both animal models and humans showed increased leukocyte infiltration after intestinal handling, which begins 3–4 h after surgery and lasts for several days (Kalff et al., 1999; Kalff et al., 2003). This suggests that although early neurogenic mechanism triggers an acute reduction in gut motor activity, the following sustained gut inflammation eventually leads to delayed postoperative dysmotility in the late phase of POI. In addition, another critical factor involved in POI is the use of analgesics, primarily of opioids after surgery. These pain-relieving drugs are able to bind to μ-opioid receptors in the GI tract, adding to the potential possibilities of POI.

**FIGURE 2:**
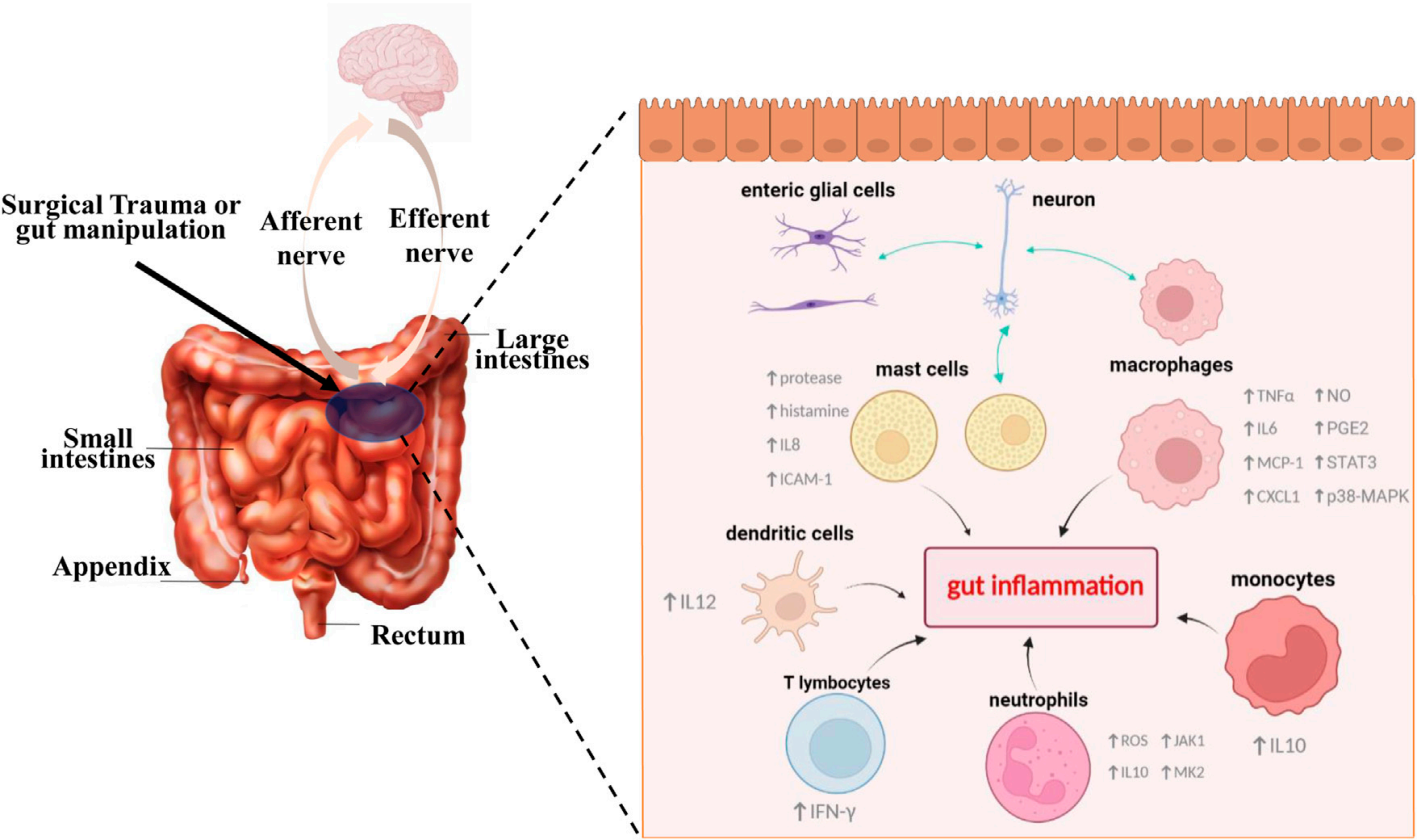
Different types of immune cells play key roles in the pathophysiologic process of POI. Surgical trauma or gut manipulation triggers the activation of different types of immune cells. In the initial stage, activated cells like mast cells and enteric glial cells can interact with enteric neurons and subsequently transmit stimulation to our brain. Meanwhile, further activation and recruitment of different immune cells such us monocytes, macrophages, neutrophils, dendritic cells, mast cells, and T lymphocytes lead to a series of gut inflammatory cascade events, accompanied by production of varied inflammatory cytokines, activation of transcription factors, and release of other active substances, all of which eventually lead to the generation and aggravation of POI.

Current approaches to prevent or treat ileus include non-pharmacological interventions and pharmacological treatments (Wattchow et al., 2021), most of which have poor therapeutic effects and lacks reliable clinical evidence (Delaney et al., 2010; Sammut et al., 2021). Hence, new strategies that target the intimate mechanisms of POI are required to complement current clinical practice and solve existing medical dilemmas (Buscail and Deraison, 2022). Recent studies have focused on the intestinal inflammation during POI, and as key components of inflammatory response, the populations of immune cells including monocytes, macrophages, neutrophils, dendritic cells, mast cells, and T lymphocytes, are proved to be closely linked to the onset of POI. Figure 2 briefly illustrates that different types of immune cells play crucial roles during POI. It is generally accepted that intestinal handling triggers the activation of resident immune cells like macrophages and neutrophils, subsequently leading to the recruitment of more immune cells. On the one hand, activation of immune cells can interact with enteric nervous system (ENS) and transmit stimulation to the spinal cord and in turn increase sympathetic output, which further inhibits myenteric neurons. Meanwhile, they trigger a serious of inflammatory events, subsequently aggravating GI dysfunction. Next, we will mainly summarize and discuss the specific roles of different immune cells in POI and relevant mechanisms implicated in the pathogenesis of this disorder.

## Immune cells in postoperative ileus

### Monocytes and macrophages

Macrophages in the GI tract constitute the major population of macrophages in the body (Mowat and Agace, 2014; Wang et al., 2019). Compared with conventional macrophages, intestinal macrophages have particular features in terms of phenotypic characterization, inflammatory response, and cytokine generation (Yip et al., 2021). It is acknowledged that intestinal macrophages are essential in mediating intestinal immunity and maintaining GI homeostasis (Bain and Mowat, 2014; Bain and Schridde, 2018; Muller et al., 2020). For paralytic ileus, research studies based on reliable animal models suggested that intestinal macrophages, primarily muscularis macrophages, play key roles in the inhibition of gut motility (De Schepper et al., 2018). These resident macrophages in the muscularis layer are activated after gut manipulation or surgical trauma, promoting the development of POI through a series of inflammatory cascade events (Grainger et al., 2017; Mazzotta et al., 2020). TNFα (Matsumoto et al., 2018), IL6 (Wehner et al., 2005), MCP-1 (Türler et al., 2002), CXCL1 (Docsa et al., 2020), and other proinflammatory cytokines or chemokines released from muscularis macrophages contribute to the recruitment of circulating leukocytes and suppress GI function by influencing intestinal muscles and nerves (Wehner et al., 2007; De Schepper et al., 2018). The production of transcription factors such as STAT3 (De Jonge et al., 2005) and p38-MAPK (Wehner et al., 2009) are also upregulated in macrophages during inflammation and are proved to be associated with POI. Moreover, muscularis macrophages induce the formation of active substances like NO, which inhibit smooth muscle *via* the activation of guanylyl cyclase (Shah et al., 2004; Francis et al., 2010). In addition, activation of macrophages results in the release of prostaglandin E2 by transient receptor potential vanilloid 4 (TRPV4) channels, which further triggers intestinal contraction (Luo et al., 2018). Taken together, muscularis macrophages take part in various inflammatory events that lead to POI, and relevant channels and pathways could be potential targets for treatment.

In contrast to resident muscularis macrophages, the infiltrated macrophages could play protective roles in POI. Farro et al. demonstrated that a population of macrophages, which was exogenously migrated and monocyte-derived, could resolve inflammation and restore intestinal motility in POI, suggesting the functional heterogeneity of different cellular origins (Farro et al., 2017). In addition, Pohl et al. pointed out the different roles of macrophages between small and large intestine. They found that progression of POI in small intestine relied on the iNOS produced by both Ly6C macrophages and Ly6C monocytes, while in colon only the latter secreted iNOS, which indicated a potential role of the intestinal microbiota (Pohl et al., 2017).

Enteric neurons are key players in macrophage-mediated inflammatory response during POI. Vagus nerve stimulation after induction of ileus has been reported to reduce the expression of inflammation-related cytokines (Stakenborg et al., 2017b). This effect depends on α7 nicotinic receptors (α7nAChR) on macrophages, leading to the regression of inflammation and amelioration of POI (Matteoli et al., 2014). In this context, many therapeutic modalities that utilize parallel mechanisms are widely studied (Cipriani et al., 2016; Yang N. N. et al., 2021). Similar to vagus nerve stimulation, the 5-HT_4_ receptor (5HT_4_R) agonist accelerates the release of acetyl choline, which subsequently activates α7nAChR on muscularis macrophages and eventually reduces the duration of POI after GI surgery (Tsuchida et al., 2011; Stakenborg et al., 2019). Considering that this effect has been confirmed in clinical trials (Gong et al., 2016), 5HT_4_R agonist can act as an effective therapeutic alternative for patients with ileus. It should be noted that not all macrophages express α7nAChR, and Stakenborg et al. proved that α7nAChR expression was restricted to M2-like pathogenic phenotype. Through culturing macrophages with myenteric ganglia, they discovered that enteric neurons contributed to the induction of α7nAChR and the install of M2 phenotype (Stakenborg et al., 2019). Therefore, further studies are required to clarify the relevant phenomena and mechanisms.

Except for a population of resident macrophages, which can sustain their numbers by cell division instead of recruiting monocytes (Mischopoulou et al., 2022), most intestinal macrophages rely on the continuous replenishment of monocytes that extravasate into GI tissue in a CCR2-dependent way (Bain et al., 2013; Bain et al., 2014), and this is also the case during POI. These CCR2-dependent monocyte-derived macrophages help restore GI function after gut manipulation (Farro et al., 2017). CCR2 knock-out mice have fewer monocyte-derived macrophages in muscularis layer due to damaged monocyte migration, which further leads to increased neutrophil-mediated immunopathology. Farro et al. observed that Ccr2^−/−^ mice show persistent muscular dysfunction and delayed GI transit recovery compared with WT mice upon intestinal handling (Farro et al., 2017), suggesting that targeting circulating monocytes and enhancing macrophage physiological repair functions could be possible strategies for reversing the symptoms of POI.

It should be specifically pointed out that IL10, mainly secreted from monocyte-derived macrophages during POI, plays an important role in the disease. Previous studies have shown that IL10 promotes polarization of macrophages and acts as a macrophage deactivator in the gut inflammation, contributing to faster recovery from ileus (Kontoyiannis et al., 2001; Stoffels et al., 2009; Makita et al., 2015). However, recent evidence suggests that IL10 leads to migration of other immune cells (also see the section on neutrophils below), which induces further inflammation (Stein et al., 2018). Indeed, Stein et al. pointed out that IL10 secreted by monocyte-derived macrophages aggravated POI, instead of relieving this sort of disorder (Stein et al., 2018).

In conclusion, the current evidence has shown that macrophages activation is crucial in the pathogenesis of POI, and that focusing on underlying molecular and cellular mechanisms may be of great use for clinical treatment. However, further studies are needed so that the observations in animal models can translate into practical therapeutic options.

### Neutrophils

Neutrophils are an indispensable component of innate immune system and act as the first kind of immune cells accumulating in large numbers at sites of inflammation (Sadik et al., 2011; Amulic et al., 2012). Neutrophils are able to release massive amounts of reactive oxygen species (ROS), produce other toxic molecules, and induce neutrophil extracellular traps during their course of reaction (Chen et al., 2021). In addition to their essential functions throughout the body, neutrophils play unique roles in intestinal homeostasis (Fournier and Parkos, 2012) and result in various pathological changes of gut disease such as inflammatory bowel disease (Zhou et al., 2018; Dinallo et al., 2019), colorectal cancer (Yang et al., 2020), and intestinal ischemia-reperfusion injury (Wang et al., 2018). Recent studies have shown that neutrophils are strongly associated with POI. Increased neutrophil infiltration can be observed in intestinal manipulation-induced models of POI, causing the induction of inflammatory mediators (Tsuchida et al., 2011; Maehara et al., 2015). Neutrophils are associated with the recruitment and activation of immune cells in the gut *via* producing cytokines such as CXCL8, IL17, and IL10 (Ferretti et al., 2003; Mantovani et al., 2011). Meanwhile, cytokines and chemokines produced by other immune cells also regulate neutrophil infiltration during intestinal inflammation (Kucharzik et al., 2005).

As highlighted above, IL10 secreted from monocyte-derived macrophages aggravates POI. Recent evidence has proved that IL10 influences neutrophil migration to traumatized sites by regulating the expression of neutrophil chemokines (Stein et al., 2018). Of note, IL-10 deficiency reduces the neutrophil extravasation into the bowel wall, and consequently ameliorates paralytic ileus. This suggests that neutrophils have direct correlation with inflammatory response and other pathological processes in POI. Interestingly, Farro et al. demonstrated that knockout of CCR2 could increase neutrophil-mediated immunopathology and prolong the clinical outcome of POI (Farro et al., 2017). These findings indicate the close relationship between neutrophils and macrophages in POI. They may have complex associations in intestinal inflammation rather than a single synergistic effect.

Janus kinase 1 (JAK1) plays an important role during inflammation and is regarded as a candidate signal pathway involved in regulating inflammatory reactions in intestinal paralysis. Sun et al. found marked activation of JAK1 after gut manipulation, accompanied by increased myeloperoxidase-stained neutrophils (Sun et al., 2019). JAK1 inhibition lowered the infiltration of neutrophils and expression of proinflammatory mediators. In addition, mitogen-activated protein kinase-activated protein kinase 2 (MK2), a downstream molecule of p38, plays an essential role in inflammation (Gorska et al., 2007). In POI, MK2 activation is upregulated, and MK2 inhibitor significantly reduces the number of neutrophils as well as the expression of proinflammatory gene (Liu et al., 2013). Moreover, the selective inhibition of p38 mitogen-activated protein kinase (MAPK) pathway leads to reduction of neutrophil infiltration after gut manipulation (Wehner et al., 2009). Taken together, signal pathways activated during POI are related to neutrophil-mediated inflammation, and from a therapeutic point of view, targeting relevant pathways may have enormous potentialities to prevent POI *via* reducing neutrophil infiltration.

### Dendritic cells

Dendritic cells (DCs) are professional antigen-presenting cells that efficiently sample the environment for foreign antigens and present them to immune system (Chang et al., 2014; Schiavi et al., 2015). In the intestine, dendritic cells are widely distributed within the lamina propria, and they are one of the immune cells central to the initiation of protective proinflammatory as well as tolerogenic immune response, which are pivotal in the maintenance of intestinal homeostasis (Persson et al., 2010; Persson et al., 2013; Worbs et al., 2017). For a long time, studies have focused on the relationship between DCs and intestinal diseases including inflammatory bowel disease and intestinal neoplasms (Bernardo et al., 2018; Yang Z. J. et al., 2021). Considering the inflammatory reaction and immune response in POI, it could be logically inferred that DCs play an irreplaceable role in the pathogenesis and development of this disease.

In fact, the intestinal DCs were observed to be activated in the mouse model of POI, with their numbers increased by 30-fold compared with sham-operated groups, which directly demonstrated the connection between ileus and this kind of immune cells (Engel et al., 2010). DCs secrete a great deal of costimulatory molecules like interleukin-12 (IL12) after surgical trauma, leading to partial activation of T_H_1-memory cells and thus stimulate intestinal macrophages to have more profound impacts on POI (Engel et al., 2010; Koscielny and Kalff, 2011). CCR7, expressed on activated DCs and T cells, is significantly upregulated after gut manipulation, and CCR7^−/−^ mice show improved intestinal muscle function in the case of surgical trauma (Koscielny et al., 2011). In addition, Pohl et al. (Pohl et al., 2017) found that CD103^+^CD11b^+^ DCs, a subset of intestinal DCs, triggered the disorder of gut motility, and that lacking such cells consequently reduced the inducible nitric oxide synthase produced by monocytes and macrophages, resulting in the amelioration of POI. As a result, human immune system plays a key role in POI, and as an important component of the immune system, dendritic cells are widely involved in the development of this disorder. Studies on various subpopulations of intestinal DCs provide us more possibilities to clearly understand their roles in POI, allowing them become a novel potential target for POI treatment.

### Mast cells

Previous evidence suggested that mast cells (MCs) are responsible for innate and adaptive immunity, neurogenic inflammation, impaired tissue function, and intestinal barrier dysfunction (Wouters et al., 2016; Traina, 2021), all of which are concerned with POI. The activation of MCs has been proved to be related to POI in both rodent models and clinical setting (De Winter et al., 2012; Berdún et al., 2015). IgE bound to the specific receptor on MCs, which triggered a series of biochemical events. Subsequently, MCs release preformed granule compounds such as cytokines, proteases, and histamine, followed by a proinflammatory response (Galli et al., 2020). MCs have bidirectional communication with nerve endings, making them able to regulate intestinal motility and organ pain (De Winter et al., 2012). In the initial phase, such interactions can be influenced by active substances secreted from MCs, which causes neurogenic inflammation and increased sensitivity. These effects on neurons eventually promote the disturbances of gut motility (Buscail and Deraison, 2022). Previous experiments on mouse models showed that gut manipulation led to mast cell degranulation and this process contributed to the development of leukocyte infiltration, implicating that MCs played a key role in the inflammatory cascade during POI (De Jonge et al., 2004). Moreover, release of mouse mast cell protease-1 (mMCP-1) in the peritoneal fluid was significantly increased after gut manipulation (Peters et al., 2015), further indicating that MCs are key players in POI. Snoek et al. used Kit^W/W−v^ and Kit^W-sh/W−sh^ mice that carry different spontaneous mutations in the gene for *ckit* and genetically lack MCs to demonstrate that absence of MCs can reduce the manipulation-induced inflammatory infiltrate and ameliorate GI transit (Snoek et al., 2012). Furthermore, the inflammatory response to intestinal handling in mast cell-deficient mice could be restored through mast cell reconstitution. In addition, evidence has shown that MCs evoke bacterial translocation to mesenteric lymph nodes and are responsible for epithelial barrier dysfunction after intestinal surgery, all of which are proved to be associated with an increased inflammatory response and delayed GI emptying, suggesting another role of MCs in the pathogenesis of POI (Snoek et al., 2012).

The relationship between MCs and POI has been also verified in clinic as well. A clinical pilot study demonstrated that the handling of intestine triggered mast cell activation and prolonged ileus in patients undergoing gynecological surgery (The et al., 2008). By quantifying mast cell activation and inflammation, the data showed that conventional abdominal hysterectomy resulted in the release of tryptase as well as an increased level of IL6 and IL8, whereas such phenomenon did not occur during minimal invasive surgery. On the other hand, intestinal manipulation-induced mast cell activation upregulated the expression of intercellular adhesion molecule-1 (ICAM-1) that is strongly associated with leucocyte recruitment (The et al., 2008). Hence, faster recovery after minimal invasive surgery may be partly owing to this, and more importantly, targeting MCs as a therapeutic approach for POI has reliable clinical proof of concept (De Giorgio and Barbara, 2008).

Mast cell stabilizers like ketotifen can reduce the release of mast cell mediators and weaken inflammation after abdominal surgery, subsequently leading to an improvement of gastric emptying (The et al., 2009; Rychter et al., 2015). In addition, evidence showed that early enteral nutrition ameliorated POI by stabilizing MCs with a cationic channel protein TRPA1 (Sun et al., 2020). Moreover, Kimura et al. found a new zinc chelator, IPZ-010, and they proved IPZ-010 caused an inhibition of inflammatory response in activated bone marrow-derived MCs, which promotes recovery of GI function after surgery (Kimura et al., 2020). Although some studies based on mouse models questioned the involvement of MCs in POI and opposed mast cell inhibitors as a therapeutic strategy for POI (Gomez-Pinilla et al., 2014), MCs are still regarded as key players in POI development considering the variation of species and current clinical evidence, and the prudent use of mast cell stabilizers could open up new perspectives for POI treatment. In addition, given the numerous differences between mucosal MCs and connective tissue MCs (Reber et al., 2015), investigations on the heterogeneity of mast cell subtypes may be valuable in POI.

### T lymphocytes

Adaptive immune system is crucial to the development of experimental POI, and as an essential cellular counterpart in this immune response, T lymphocyte is closely linked to the pathological process of this disorder. T lymphocytes are widely known to exist in human blood and lymphoid tissue, resident in the gut at the same time (Ma et al., 2019). They have long been observed interacting with gut microbiota to regulate intestinal homeostasis (De Oliveira et al., 2017; Caruso et al., 2020). So far, studies on T lymphocytes, especially helper T cells, have given us a deeper insight into the molecular and cellular mechanisms involved in POI.

Gut manipulation retarded the transit of orally administered fluorescent dextran in mice, while CD4 knockout mice eliminated such delay and lightened the local inflammatory response, implicating that CD4^+^ T helper cells are critical factors involved in POI (Engel et al., 2010). Nevertheless, this effect may be related to the type of T helper effector cells. As a subtype of CD4^+^ T cells, T_H_1 cells can be induced mainly by IL-12, and subsequently secrete a variety of cytokines, thereby mediating the cellular immune response and participating in the inflammatory response. Koscielny et al. identified that surgical trauma and local inflammation trigger the release of IL-12, leading to production of large numbers of interferon-γ (IFN-γ) by activated T_H_1-memory cells, which consequently enhances the inflammatory process underlying POI and causes GI hypomotility by promoting intestinal macrophages to secrete NO (Koscielny and Kalff, 2011). These findings present us a close connection between T-helper type 1 cell-mediated adaptive immune response and macrophage-mediated innate immune system during POI, assisting us to discover a fire-new way to reduce the duration of POI.

In addition, activated T_H_1 cells at surgical trauma sites can migrate to unmanipulated intestinal segments through the bloodstream, subsequently disseminating ileus over the entire intestinal tract (Engel et al., 2010). Immunosuppressive FTY720 or inhibition of IL-12 can block the T_H_1 cell exit to the portal vein blood and thus prevent POI. These findings provided further evidence that T_H_1 cells are major participants in POI and play irreplaceable roles in its progression.

Despite the well-known role of T_H_1 cells in POI, T_H_2 cells may have an ignorant effect on the disease progression. In fact, recent studies have proved that POI is related to an increase in both T_H_2 cytokines and T_H_2 cells, accompanied by an increased number of mast cells as well as upregulated IgE and histamine plasma levels. This T_H_2 response could be linked to the ROS-mediated activation of NF-κB and p38 MAPK signaling pathways (Lin et al., 2021). In summary, T lymphocyte-mediated immune response has been identified to be a crucial target in the pathological process of POI, but the characteristics of such immune response are not completely understood. Hence, further research is needed to help develop the comprehension of POI to a brand-new phase.

## Other important contributors

An emerging cellular target in the field of neurogastroenterology and GI disorders is the enteric glial cell that constitutes a crucial part of the enteric nervous system and maintains intestinal homeostasis through interactions with resident immune cells and other cell types (Sharkey, 2015; Yoo and Mazmanian, 2017). Enteric glial cells play a pivotal role in normal gut motility, and disruption of the balance maintained by this cell population consequently leads to motility disorders and GI diseases (Gulbransen and Christofi, 2018; Seguella and Gulbransen, 2021). Notably, studies have revealed that both finger manipulation and high pneumoperitoneum pressure during intestinal surgery cause abnormal mechanical forces on the gut and its mesentery, activating enteric glial cells and converting them to a pathogenic state referred to as a reactive glial phenotype that directly contributes to POI (Mazzotta et al., 2020). Recently, Schneider et al. found that surgical trauma triggers ATP release which further induces a reactive glia phenotype known as “gliosis” (Schneider et al., 2021). The induction of enteric gliosis through ATP depends on the p38-MAPK signaling pathway, and this process subsequently leads to intestinal inflammation and impaired gut motility in POI. Furthermore, P2X2, a relevant ATP receptor, is demonstrated to be linked to ATP-induced enteric gliosis and inflammation. Therefore, blocking enteric glial P2X2 receptors could be a potential therapy in ameliorating POI.

Interstitial cells of Cajal (ICC) are proved to be another contributor to the genesis of POI. Known as intestinal pacemaker cells, ICCs play a significant role in regulating GI motility (Sanders et al., 2014). Kaji et al. demonstrated that the production and propagation of pacemaker potentials *via* ICCs were disrupted through a nitric oxide pathway, further resulting in GI dysmotility after intestinal manipulation (Kaji et al., 2018). This pathological change will be ameliorated as the intestinal inflammation subsides. Hence, it is reasonable to infer that nitric oxide synthase inhibitor may have therapeutic potentials for POI by suppressing the disruption of ICC networks. Moreover, studies have shown that acupuncture protects ICCs in rat models of POI, leading to amelioration of GI function (Deng et al., 2017; Deng et al., 2019). Of note, intestinal mesothelial cells in the abdominal cavity are also involved in the development of POI as the inflammatory response mediated by them is a significant mechanism in many clinical conditions including gut dysmotility (Mihara et al., 2017). Relevant anti-inflammatory pathways regulated by α7nAChR expressed on intestinal mesothelial cells may have a therapeutic potential through connectivity with enteric nerves. In addition, other intestinal components like microbiome contribute to POI as well, making POI a complex pathological process.

## Conclusion and future perspectives

POI is an unsolved clinical problem that demands further investigation to find a novel therapy. Recent studies have focused on the immunological and inflammatory response during POI, and comprehensive understanding of relevant cellular mechanisms provides a promising target for POI treatment. As key components of this process, populations of different immune cells are closely related to POI development. Strategies that target macrophages or MCs are already proven to be effective in clinical setting, but they still need further evidence. Our review provides a comprehensive understanding of different types of immune cells in the development of POI, with emphasis on molecular and cellular mechanisms. Although most of their roles have been elucidated in previous studies, the complex interactions between these players are still poorly understood. More promising and effective therapies are likely to evolve from a deep comprehension of relevant mechanisms underlying POI.
